# Childhood adversity subtypes and depressive symptoms in early and late adolescence

**DOI:** 10.1017/S0954579414000625

**Published:** 2015-08

**Authors:** Michelle C. St Clair, Tim Croudace, Valerie J. Dunn, Peter B. Jones, Joe Herbert, Ian M. Goodyer

**Affiliations:** a University of Cambridge; b University of York

## Abstract

Within a longitudinal study of 1,005 adolescents, we investigated how exposure to childhood psychosocial adversities was associated with the emergence of depressive symptoms between 14 and 17 years of age. The cohort was classified into four empirically determined adversity subtypes for two age periods in childhood (0–5 and 6–11 years). One subtype reflects normative/optimal family environments (*n* = 692, 69%), while the other three subtypes reflect differential suboptimal family environments (aberrant parenting: *n* = 71, 7%; discordant: *n* = 185, 18%; and hazardous: *n* = 57, 6%). Parent-rated child temperament at 14 years and adolescent self-reported recent negative life events in early and late adolescence were included in models implementing path analysis. There were gender-differentiated associations between childhood adversity subtypes and adolescent depressive symptoms. The discordant and hazardous subtypes were associated with elevated depressive symptoms in both genders but the aberrant parenting subtype only so in girls. Across adolescence the associations between early childhood adversity and depressive symptoms diminished for boys but remained for girls. Emotional temperament was also associated with depressive symptoms in both genders, while proximal negative life events related to depressive symptoms in girls only. There may be neurodevelopmental factors that emerge in adolescence that reduce depressogenic symptoms in boys but increase such formation in girls.

Adolescence is a time of many developmental changes, and rates of both depressive symptoms and disorder start to increase from early adolescence, usually in midpuberty (Angold, Costello, & Worthman, 1998; St Clair et al., 2012). Although this occurs in both genders, it is especially pronounced in adolescent girls (Angold et al., 1998). Research has identified many psychosocial risk factors behind this increase in depressive symptoms during adolescence. These include distal factors (childhood adversity/maltreatment or temperament/personality), cognitive biases (negative inferential style or negativity biases), and proximal upsetting life events (Hankin & Abramson, 2001). Key among the distal factors are adversities in the childhood years involving physical, sexual, or emotional maltreatment and severely discordant family relations. While there are many studies demonstrating clear-cut associations between adversities and later depressions emerging in adolescence and young adulthood, the pathways accruing longitudinally from distal childhood adversities that lead to psychopathology remain unclear. In particular, it is not known whether childhood adversities exert direct effects on adolescent well-being that require no further exposure to stressful experiences or operate as prior vulnerabilities with their latent effects revealed only in the presence of more proximal stressors.

Fraley and colleagues (Fraley, Roisman, & Haltigan, 2013; Haltigan, Roisman, & Fraley, 2013) formalized two distinct paths from childhood adversities to later behavior. The first path is an enduring effects model, which indexes a direct relationship between early experiences and later cognitive and behavioral outcomes. The second path is a revisionist model, where the effects of early experiences reduce throughout development until there is no predictive value. Whether these models are applicable and have validity for psychopathology outcomes in adolescence has yet to be examined.

As well as the nature of early adversities, recent reports have begun to consider the importance of the timing of early experiences and their putative impact on behavior (Evans et al., 2012; Narayan, Englund, & Egeland, 2013). Introducing this concept evokes a role for maturation influencing sensitivity to the proximal environment over the child and adolescent years. This differential influence of maturation can be tested by determining whether the age of exposure to adversity influences the behavioral outcome of interest.

A third theoretical component is the nature of exposure model, whereby the impact of the social environment is dependent on the duration and severity of exposure. In support of this model are the replicated findings that the manifestations of depressive symptoms (i.e., symptom counts, latent dimensions, or categories reflecting presence of disorders) increase with severity of exposure to childhood adversities occurring before the age of 11 (Espejo et al., 2006; Hammen, Henry, & Daley, 2000; Hazel, Hammen, Brennan, & Najman, 2008; Kendler, Kuhn, & Prescott, 2004; McLaughlin, Conron, Koenen, & Gilman, 2010). This quantitative model implicates a possible dose–response relationship between number and severity of adversities and likelihood of a depressive outcome, even if this effect is nonadditive and complex (Brewin, Andrews, & Valentine, 2000; Fergusson, Boden, & Horwood, 2008; Gilbert et al., 2009; Li, Ahmed, & Zabin, 2012; Widom, DuMont, & Czaja, 2007).

There may be a relatively straightforward linear tipping point whereby risk is elevated and symptoms emerge regardless of the nature or timing of social experiences. Whether this would be revealed for rises in depressive symptoms with the inclusion of distal environmental risks has yet to be adequately tested. Examination of such an effect would need to take into account the well-established notion that environmental adversities frequently co-occur and collectively exert nonadditive effects on subsequent risk for mental illness (Berzenski & Yates, 2011; Green et al., 2010; Shanahan, Copeland, Costello, & Angold, 2011).

As well as environmental adversities, within-subject factors are likely to shape the developmental paths and the overall liabilities for increases in depressive symptoms over adolescence. A key candidate is the childhood temperamental style of emotionality contributing to the likelihood of making negative cognitive inferences following exposure to and processing of undesirable life events (Abramson, Alloy, & Metalsky, 1989; Beck, 1987; Hammen, 2006; Hankin & Abramson, 2001). Whether there is a direct “long reach” or a “vulnerability” effect via mediating experiences for this temperamental predisposition on the risk for increasing depressive symptoms during adolescence is not clear. Such a within-subject factor may also alter the effect of exposure to adversities by increasing the overall sensitivity to the environment over time and/or moderating the general negative burden of adversities accruing in the childhood years.

In addition to gender differences in depressive symptoms (e.g., St Clair et al., 2012), girls report experiencing more upsetting life events (e.g., Ge, Lorenz, Conger, & Elder, 1994; Rudolph & Hammen, 1999), higher levels of negative emotionality or neuroticism (Clark, Steer, & Beck, 1994; Goodyer, Ashby, Altham, Vize, & Cooper, 1993; Watson, Gamez, & Simms, 2005), and may be more likely to have cognitive biases (Hankin & Abramson, 2002). Overall, parents discuss emotions and feelings more with girls than with boys (Adams, Kuebli, Boyle, & Fivush, 1995), thus socializing girls to think about and potentially experience their emotions more than boys, which may contribute to the well-known gender differences in depressive symptoms (St Clair et al., 2012). In addition, this gender difference may be exacerbated because of the higher rates of exposure to childhood sexual abuse in girls (Rind, Tromovitch, & Bauserman, 1998). It is apparent that gender differences may be found in the predisposing factors behind depression well before the emergence of measurable gender differences in depressive symptoms by adolescence (Hankin & Abramson, 2001).

Focusing specifically on the emergence of gender-differentiated depressive symptoms during the adolescent years, there are four components of a developmental model that can be addressed with population-based prospective repeated measures. These components are whether (a) the impact of early childhood adversities on adolescent symptoms persist or reduce over time, (b) the age of the child when exposed to adversity influences the long-term effects of such experiences, (c) adolescent maturation per se contributes to changes in depressive symptom levels and sensitivity to the environment, or (d) distinct gender differences in predisposing depressogenic factors in childhood potentially underpin the expected differences in depressive symptoms by adolescence.

In this paper we used the ROOTS cohort, a prospective investigation of adolescent mental health and development (Goodyer, Croudace, Dunn, Herbert, & Jones, 2010) to investigate the relative contributions of childhood adversities, negative emotionality, and more recent life events on adolescent depressive symptoms for each gender. We addressed the exposure to family-focused risks in childhood by conducting interviews with parents of 14-year-olds to capture maltreatment experiences, discordant marriages, disinterested, punishing and conflict laden parenting styles, financial hardships, and poverty. This aimed to create an objective data-driven measure of the family environment within which the child was reared between infancy and entering secondary school. We used a person-centered rather than a variable-centered approach to reveal classes of adolescents exposed to particular patterns of multiple childhood adversities (Dunn et al., 2011). This makes use of the patterning of co-occurrences for different adverse family environments to classify individuals to unique family subtypes that might be etiologically and prognostically distinct (Copeland, Shanahan, Costello, & Angold, 2009; Dunn et al., 2011; Menard, Bandeen-Roche, & Chilcoat, 2004; Parra, DuBois, & Sher, 2006). We were then able to establish putative causal pathways between distal exposure to family-based childhood adversities, negative emotionality and proximal negative life events, and self-reported depressive symptoms recorded at 14 and 17 years of age, which is within the highest period of risk for increasing levels of depressive symptoms.

One objective of this research was to determine if a direct (enduring type) model accounts for the effects of early experiences on later depressive symptoms. To achieve this, we used the aforementioned empirical typology of family environments across two different time periods in the first 11 years of life (“early”: birth to approximately 5 years; and “later”: approximately 6–11 years) and self-reported depressive symptoms at both 14 and 17 years of age. Another objective was to determine whether an indirect (revisionist type) model might also be present and account for alterations in later depressive symptoms. This was determined by including parent-reported negative childhood emotionality and recent life events retrospectively recalled by adolescents as potential modulators of any effects of childhood adversity on depressive symptoms within the adolescent years. We hypothesize that childhood adversity, potentially in combination with emotional temperament, may facilitate a chaining of events, leading to increased rates of life events that further increase the risk of depressive symptoms later in life. Finally, we examined whether age of exposure (early versus later childhood adversities) altered the findings, specifically focusing on whether the paths were altered if exposure was in the preschool or the school-age years. This was done for boys and girls separately, and then pathways were compared to determine any significant gender-differentiated paths.

## Methods

### Participants

We studied 1,005 adolescents (boys = 409, girls = 596), comprising 81% of the 1,238 adolescents (average age = 14.5 years, *SD* = 3.38 months) from the East of England recruited as part of the ROOTS cohort study (Goodyer et al., 2010). A follow-up was conducted 36 months after study entry (average age = 17.5, *SD* = 4.06 months). Measures of the social environment, depressive symptoms, and clinical disorders were obtained using postal questionnaires and face-to-face interviews at both testing waves. Participants were excluded from analysis when they either had no valid data on childhood adversity and negative emotionality or had missing data on all adolescent variables (*n* = 124 of 1,238). In addition, participants determined to be prepubertal at study entry were excluded because research indicates that depressive symptoms and affective disorders markedly rise during puberty and also that the relationship between life events and symptoms may also change once adolescents enter puberty (Angold et al., 1998; Euling et al., 2008; Rudolph & Flynn, 2007; St Clair et al., 2012). Therefore, we further excluded 100 prepubertal boys and 9 prepubertal girls (see Supplementary Materials for the definition of pubertal status). Written and informed consent was obtained from all participants and their parents in compliance with local ethical approval.

### Measures

#### Childhood adversity

Childhood adversity was assessed using the Cambridge Early Experiences Interview (Dunn et al., 2011), a semistructured respondent-sensitive interviewer led procedure that collected retrospective accounts of the quality of family environment in three time domains of childhood: early childhood (preschool years: birth to the start of primary school, approximately 5 years of age), later childhood (primary school years: approximately 6–11 years of age), and early adolescence (early secondary school: approximately 11 years, age of interview). Prior to the interview, the main caregiver was sent timelines of each time domain and asked to populate them with significant events in his or her child's life. Because these timelines were delineated by major changes in the child's life (start of primary school and start of secondary school) rather than simply ages (age 5 or 11), we are confident we captured events within the correct time period to as high a degree of accuracy as possible with retrospective interviews (White, Armstrong, & Saracci, 2008). In addition, sending the timelines to parents to fill in before the interview allowed parents to consult their records and photographs to accurately place events on the timeline.

The interview consisted of 26 cue questions and was carried out with the main caregiver in the family home. These items measured their perceptions of the family environment, specifically inquiring about family relationships; economic circumstances; health of family members; physical, sexual, and emotional abuse; crime; and chronic social impairments. An example of a cue question is “Were there any times when you didn't feel affection for [child's name] . . . ? When you couldn't show affection?” A separate categorical rating for each cue questions was made. Lifetime psychopathology for all family members (including step family members and partners resident over 6 months) was assessed by the main caregiver using the Mini-International Neuropsychiatric Interview Mental State Interview (Sheehan et al., 1998).

The same questioning procedure was applied for each of the three time periods in order to establish a best estimate of the timing of family experiences. Reference was made throughout the interview to the completed timeline. Recorded adversities were assigned an impact rating by the interviewer determined by whether the experience was likely to have a moderate or severe negative effect on the family environment lasting more than a few days. The focus was to determine whether individuals were living in an optimal or suboptimal family environment that would enhance or detract from the personal and social development of family members (Dunn et al., 2011).

A person-centered approach using model-based cluster analysis techniques can distinguish individuals who would otherwise be indistinguishable if grouped by a simple sum score or “count of the number of adversities” (Copeland et al., 2009; Menard et al., 2004; Parra et al., 2006). Such an empirical approach may reveal subtypes with differing etiological mechanisms and prognostic outcomes not readily observable using a variable-centered analysis. Therefore, latent class analysis (LCA), a latent structure/mixture model, was used to subtype individuals by their exposure to multiple adversities (as reported in the Cambridge Early Experiences Interview). Analyses were performed within each of the three time domains separately.

Overall, the 26 items grouped into nine statistically distinct adversity descriptors that contributed to the overall quality of the family environment: (a) family loss, (b) family discord, (c) overt abuse and parental criminality, (d) financial problems including unemployment, (e) paternal psychiatric illness, (f) maternal psychiatric illness, (g) paternal parenting style, (h) maternal parenting style, and (i) maternal lack of affection or engagement. For overt abuse, the most common perpetrators were the child's father or stepparent.

**Figure 1.**
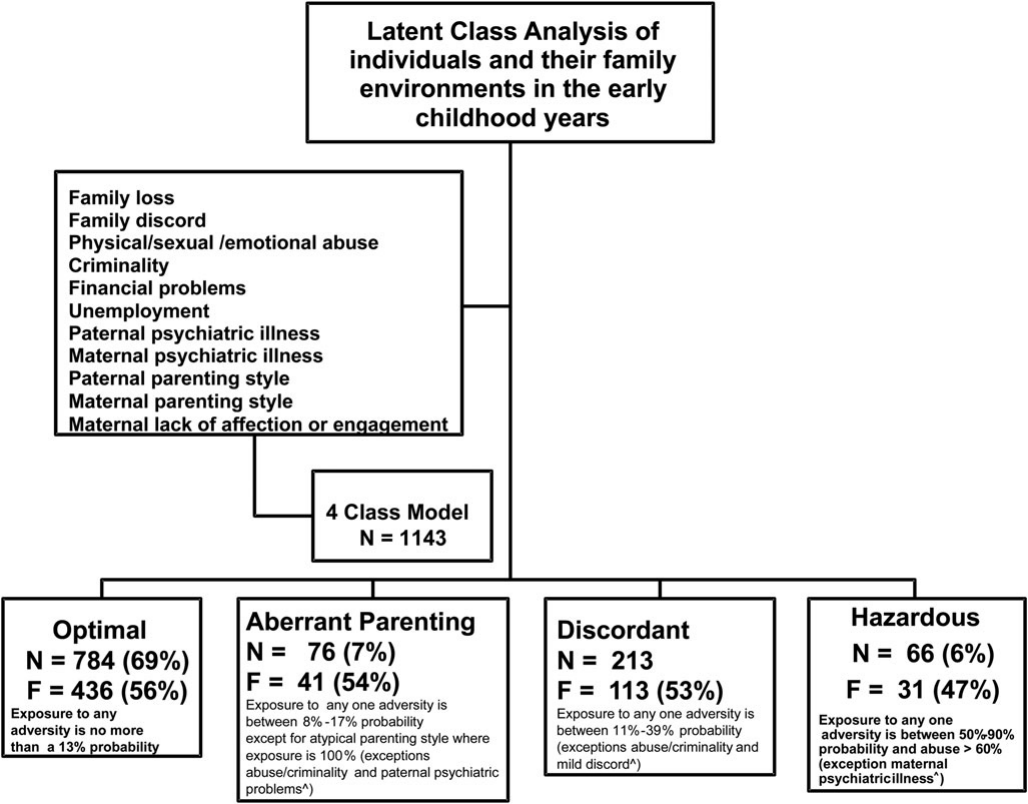
Description of four classes of adolescents derived from the latent class analysis fully reported by Dunn et al. (2011). 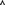
Exceptions are the following: aberrant parenting, no abuse/criminality and only 3% paternal psychiatric problems; discordant, only 2.4% abuse/criminality and 47% mild discord; hazardous, 35% maternal psychiatric problems; 33% lack of maternal affection; 46% parental divorce/loss. The latent classes described above were labeled as follows in Dunn et al. (2011): low (optimal), atypical parenting (aberrant parenting), moderate (discordant), and high (hazardous).

Using these nine descriptors, the LCA revealed that a four-class model of individuals adequately summarized the data for each of the three time domains (see Figure 1). The proportion of individuals exposed to each adversity varied between classes, with the exception of overt abuse and criminality and unpredictable/atypical parenting behaviors by *both* parents. Both these exceptions were only present in one class each, confirming qualitative differences in the nature of family environments between the classes. The four classes were also clearly distinct from each other based on the probabilistic level of exposure to one or more of these family environmental adversities.

There was one class whose characteristics were considered as an optimal or normative family environment, with the other three reflecting suboptimal family environments that might convey psychiatric risk. See Dunn et al. (2011) for full details of the LCA.

Below are descriptions of the four classes, with prevalence rates for the early childhood time window (0–5 years) used to illustrate the differences between the classes. A similar pattern was found for the remaining two time windows. We labeled the four classes as follows:

*Optimal class:* The largest subtype of adolescents (784, 69%) were those in a class with less than a 13% probability of being exposed to each indicator of adverse family environments. Adversity rates were only above 10% for financial difficulties and unpredictable/atypical paternal parenting style. We termed this the optimal class because of the overall likelihood that personal growth and the integrity of the family system would be enhanced and maintained, and be associated with a low risk for poor health outcomes in individual family members.
*Aberrant parenting class:* This suboptimal class (76, 7%) had a somewhat elevated probability (8%–17%) of exposure to most of the adversities but uniquely carried a 100% and >70% probability of inconsistent/atypical maternal and paternal parenting, respectively. We used the term aberrant parenting to convey the qualitative nature of exposure in this class. In addition, there was no report of abuse/criminality and a low rate of paternal psychiatric problems (3%). Individuals in this class were 1.4 times more likely than the optimal class to report any psychiatric diagnosis by age 14 (Dunn et al., 2011).
*Discordant class:* This class (213, 19%) is the largest of the suboptimal classes and consisted of individuals with a clear-cut increase in the probability (11%–39%) of being exposed to each of the indicators of family adversity, with the exception of a very low rate of overt abuse/criminality (2.4%) but a higher rate of family discord, at 47%. The term discordant is used to convey the higher presence of family/marital disagreements within family life than in other classes. The observed prevalence rate of mental illness by 14 years of age is just over twice as great (2.3 times) when compared to the optimal class (Dunn et al., 2011).
*Hazardous class:* The smallest suboptimal class (66, 6%) had a high probability of between 50% and 90% of exposure to most of the family adversities, including very severe family discord, and uniquely a >60% of experiencing direct maltreatment and/or parental criminality. However, both maternal psychiatric problems and maternal affection had slightly lower rates of endorsement (<33%) as well as a lower rate of family loss (46%). We termed this class hazardous because the average rate of adversity (including abuse to the offspring) was very high, indexing an environment that is both unpredictable and potentially dangerous to family members. The likelihood of mental illness by 14 years of age is four times more likely compared to the optimal class (Dunn et al., 2011).

These classes were created for all time periods using the same parameters. Individuals could be in the same or different classes over all time periods. Changes in class membership within childhood are reported in the Results section. The descriptions above for the early adversity time period hold for the later time periods as well. In this study, we have only used the first two time periods 0–5 and up to age 11 to focus the findings on the role of childhood adversities occurring prior to the measurement of depressive symptoms, life events, and disorders.

#### Emotionality, Activity, and Sociability Temperament Questionnaire

Negative emotional temperament was assessed by the parents at the initial assessment when the adolescents were aged 14. This was measured using the 20-item parental-report Emotionality, Activity, and Sociability Questionnaire (EAS; Buss & Plomin, 1984), and parents rated each item based on how typical it was of their child. We only considered the emotionality subscale (5 items) because only this domain of the EAS has been shown to be related to depressive symptoms and episodes of major depressive disorder in adolescence (e.g., Goodyer et al., 1993). Internal consistency reliability estimates for this subscale were high, despite its short length: α = 0.81 for boys and girls separately, with a pooled estimate of α = 0.81.

#### Recent Life Events Questionnaire

A self-report questionnaire evaluating major life events over the previous 12 months (Goodyer, Herbert, Tamplin, & Altham, 2000) was administered to the adolescent at ages 14 and 17. The specific questions were about the following events: changing schools, changes in family composition, moving house, disasters at home (fire, flood, or burglary), serious illness (either the student, family, or close friends), time spent in hospital (either the student, family, or close friends), deaths, loss of family pet, lost touch with friends, problems with friendships, or any other events. Reported events were self-rated as pleasant or unpleasant (very pleasant, pleasant, neither, quite unpleasant, or very unpleasant) as well as whether the event impacted them for 2 weeks or more. An event was considered a “significant undesirable life event” if rated as being quite or very unpleasant, which was likely to influence them negatively for 2 weeks or more.

#### Moods and Feelings Questionnaire

The self-report Moods and Feelings Questionnaire is a 33-item self-report instrument that was developed to measure current (last 2 weeks) depressive symptoms in 8- to 18-year-olds (Costello & Angold, 1988). The instrument has excellent internal consistency reliability (at age 14, α = 0.89 for boys and α = 0.93 for girls, pooled α = 0.92; at age 17, α = 0.92 for boys and α = 0.93 for girls, pooled α = 0.93) and criterion validity for depressive episodes in adolescents (Daviss et al., 2006). We used a four-category response version (never, sometimes, mostly, or always), but collapsed to three response categories (never, sometimes, or mostly/always) for analysis because there were very sparse endorsement rates for the option “always.” This gives sum scores ranging from 0 through to 66, with the higher the score, the greater the level of depressive symptoms. This measure was completed at both time points by the adolescents, and sum scores were generated from all items.

#### Episodes of Psychiatric Disorder (Schedule for Affective Disorders and Schizophrenia for School-Age Children—Present and Lifetime Version)

At ages 14 and 17 years all adolescents were interviewed face-to-face for present and lifetime episodes of psychopathology using sections of the Schedule for Affective Disorders and Schizophrenia for School-Age Children—Present and Lifetime Version relevant for common mental illnesses in the community (depressive disorders, anxiety disorders, obsessive compulsive disorders, eating disorders, conduct and oppositional disorders, or substance and alcohol misuse and dependence) to generate DSM-IV axis 1 diagnoses. Interviews were conducted by fully trained research assistants, and diagnoses were reached at consensus meetings with senior staff, including adolescent psychiatrists. There was no test–retest undertaken with the adolescent. Agreement of diagnosis between raters at consensus meetings was >95% before discussion.

### Data analysis and modeling strategy

We profiled and related the level of depressive symptoms at 14 and 17 years by all four adversity classes for the early and later childhood time periods. Linear regression was used to determine any differences between symptom levels within the classes for each gender separately. A robust sandwich estimator was used to accommodate both unequal variances across the adversity classes and the skewed distribution in depressive symptoms. We used ordered logistic regression to evaluate the differences in life events across the adversity classes and genders. These analyses were conducted in Stata 12 (StataCorp, 2011).

Using path analyses, specified as structural equation models, we subsequently revealed the longitudinal relationships between exposure to the four childhood adversity classes and depressive symptoms reported at 14 and 17 years. Although the importance of the distal risk factor of early childhood adversity is in no doubt, we wished to evaluate whether the direct association was retained when combining several risk factors jointly in predicting subsequent depressive symptoms. In order to test the influence of a within-subject factor on both the impact of childhood adversities and the emergence of depressive symptoms, negative emotionality was included in the analyses. We conducted similar models with later childhood adversity. The adversity predictor variable was inputted as all four adversity classes in the order described above.

Although we conducted two separate models for early and later childhood adversity, we do not try to claim that adversity up to age 5 is entirely separate from adversity in the primary school years. However, from a developmental perspective, we view the effect of early childhood adversity as slightly less dependent on later childhood adversity than later childhood adversity is on early childhood adversity. Because both time periods are inherently related to each other and data is collected from a single respondent at the same retrospective interview, we have included a covariate within each model. For the early childhood adversity models, we use a binary covariate that indicates whether individuals moved from a class with less adversity in early childhood to a class with more adversity. For later childhood adversity models, we used a binary covariate as to whether or not there was adversity in early childhood (optimal vs. aberrant parenting, discordant, and hazardous).

All models were estimated using Mplus version 6.1 (Muthén & Muthén, 1998–2011). The models for boys and girls differed, but they were estimated simultaneously using the GROUPING command (WLSMV estimator). Model fit evaluations were evaluated (root mean square error of approximation, comparative fit index, and Tucker–Lewis index). To test whether paths were significantly stronger in one gender or across time, we saved the reference model (described in the results) and ran a comparison model with specific paths constrained, either across time (association between life events and depressive symptoms) or between the genders. Testing whether the associations between life events and subsequent Moods and Feelings Questionnaire differed from early to later adolescence was done within each gender separately.

We used the MODEL INDIRECT command to test for indirect paths between childhood variables (adversity and negative emotionality) and age 17 outcome variables (recent life events and depressive symptoms). Standard errors were calculated using a bootstrap approach (10,000 resamples). We tested for all indirect paths, which is consistent with recent mediation literature (MacKinnon, Krull, & Lockwood, 2000; MacKinnon, Lockwood, Hoffman, West, & Sheets, 2002). Testing for all indirect paths allows us to reveal potential suppressor effects that would not be revealed if we only tested for indirect effects when there was a significant main effect (i.e., the Baron and Kenny approach; Baron & Kenny, 1986).

The childhood adversity and negative emotionality exogenous variables were allowed to covary within all models. All models included whether there was a diagnosis of an affective disorder before age 13 as a binary covariate on all the outcome variables (depressive symptoms and life events), because the evidence indicates that depressive disorders may alter the future relationship between life events and depressive symptoms (Hammen, 1991). We chose age 13 because this was prior to the first possible measurement of a significant, negative life event. We also conducted control models removing any individual who reported any affective disorder between ages 13 to 14 or 16 to 17 (the years of interest in the current analysis) to determine if any findings related to depressive symptoms were simply accounted by a recent depressive illness. Further control models investigated what specific contribution each adversity class made to the path analysis results. See the online only Supplementary Materials for further details of the path models and fit statistics.

### Objectives

We determined whether the distal influence of childhood adversity reduces over time or is maintained across adolescence. In addition, we evaluated whether childhood adversity may set off a chaining of events with other risk factors for depression, particularly with negative emotionality and adolescent-reported proximal life events. Thus, we investigated within path analysis whether the long-term effect of childhood adversity to later maladaptive outcomes at ages 14 and 17 is modulated through complex etiological pathways. The possibility of different long-term effects may depend on when the distal childhood adversities occurred (before 5 or between 5 and 11 years), so models were run testing the effects of each of the two time periods. Within all of these analyses, we evaluated boy and girl adolescents separately, because we assumed the etiological pathways to increased depressive symptoms may differ between the genders.

The four key hypotheses tested are whether

1. there are statistically significant nonlinear differences in levels of depression symptoms between the optimal and suboptimal subtypes at both 14 and 17 years of age;
2. the associations between childhood adversity and depressive symptoms maintain or reduce with age, potentially dependent on the timing of adversity in childhood;
3. proximal life events in adolescence are significant contributory negative experiences leading to depressive symptoms in the presence of distal childhood adversities and the temperamental disposition of emotionality; and
4. there are gender-differentiated longitudinal pathways that provide an explanatory framework for higher depressive symptoms in females compared to males.

## Results

### Characteristics of the sample

Of the 1,005 adolescents in the study, 535 (53%) were exposed to optimal family environments during the whole of childhood, while 470 (47%) were exposed to suboptimal family environment at some point in childhood and were at increased risk for mental illness. As expected, for the full sample, girls reported higher depressive symptoms at both ages, β = 3.97, 95% confidence interval (CI; (2.79, 5.16), *p* < .001 at 14 and β = 3.60, 95% CI (2.16, 5.05), *p* < .001 at 17. Correlations were computed between ages 14 and 17 depression symptom scores, demonstrating expected significant associations over time for both for boys (*r* = .44, *p* < .001) and girls (*r* = .46, *p* < .001). The moderate level of correlations may be (a) simply reflecting the ebb and flow of symptom levels across time or (b) because of other factors being responsible for individual-level change in depressive symptoms across adolescence.

### Adversity class membership and self-reported depressive symptoms at 14 and 17 years

Descriptive statistics of depressive symptoms at ages 14 and 17 for the early and later childhood adversity classes are shown in Table 1.

**Table 1.** Means (SD) for the Moods and Feelings Questionnaire in adolescent boys and girls at age 14 and 17 within each of the early and later childhood adversity classes with adversity class comparisons between the adversity classes provided

Early Childhood Adversity					
Adolescent Boys	Optimal (*n* = 275)	Aberrant Parenting (*n* = 30)	Discordant (*n* = 75)	Hazardous (*n* = 29)	Comparisons
Age 14	12.26 (8.09)	12.18 (8.30)	13.96 (7.90)	17.04 (9.30)	H > O: β = 4.77, *p* < .05 H > A: β = 4.86, *p* < .05
Age 17	11.94 (10.07)	9.64 (7.54)	12.36 (8.35)	14.76 (7.44)	*ns*
Later Childhood Adversity					
	Optimal (*n* = 243)	Aberrant Parenting (*n* = 26)	Discordant (*n* = 91)	Hazardous (*n* = 49)	
Age 14	12.14 (8.27)	10.42 (4.84)	14.16 (7.52)	15.67 (9.82)	D > O: β = 2.01, *p* < .05 H > O: β = 3.53, *p* < .05 D > A: β = 3.74, *p* < .005 H > A: β = 5.25, *p* < .005
Age 17	11.46 (9.37)	8.26 (7.20)	12.94 (10.45)	16.23 (8.43)	H > O: β = 4.77, *p* < .005 H > A: β = 7.96, *p* < .001 D > A: β = 4.68, *p* < .05
Early Childhood Adversity					
Adolescent Girls	Optimal (*n* = 417)	Aberrant Parenting (*n* = 41)	Discordant (*n* = 110)	Hazardous (*n* = 28)	
Age 14	16.13 (10.51)	17.0 (8.72)	17.61 (9.47)	25.6 (10.94)	H > O: β = 9.47, *p* < .001 H > A: β = 8.60, *p* < .005 H > D: β = 7.99, *p* < .005
Age 17	15.10 (10.57)	15.78 (10.86)	16.5 (11.82)	21.83 (7.52)	H > O: β = 6.74, *p* < .001 H > A: β = 6.06, *p* < .05 H > D: β = 5.33, *p* < .05
Later Childhood Adversity					
	Optimal (*n* = 352)	Aberrant Parenting (*n* = 31)	Discordant (*n* = 163)	Hazardous (*n* = 50)	
Age 14	15.34 (9.88)	19.37 (10.99)	18.41 (10.27)	21.52 (12.06)	A > O: β = 4.02, *p* = .05 D > O: β = 3.06, *p* < .005 H > O: β = 6.18, *p* < .005
Age 17	14.29 (9.79)	17.69 (14.25)	17.26 (11.18)	19.16 (12.79)	D > O: β = 2.97, *p* < .01 H > O: β = 4.87, *p* < .05

#### Boys

In adolescent boys exposed to early childhood adversities (0–5 years), mean depression scores at age 14 were significantly increased in the hazardous compared to the optimal and the aberrant parenting classes. The discordant subtype was not different from other classes on depression scores. However, by 17 years of age, this effect had diluted: depressive symptoms did not significantly differ between any of the four adversity classes. In contrast, later childhood adversities (5–11 years) showed that at 14 years there were two adversity classes, hazardous and discordant, with elevated depression scores when compared to both the optimal and aberrant parenting classes. However by age 17, the later childhood hazardous class only continued to have elevated levels of depressive symptoms when compared to both the optimal and the aberrant parenting classes. The discordant class had increased depressive symptoms at age 17 only when compared to the aberrant parenting class.

#### Girls

For girls, the early childhood adversity hazardous class showed elevated depression symptoms at 14 years of age when compared to the remaining three early adversity classes. In contrast to boys, by age 17 this pattern remained unchanged. However, exposure to later childhood adversities was associated with significant increases in depression scores at 14 years for all three suboptimal classes when compared to the optimal class. By 17 years, the pattern changed, with elevated symptoms only in the hazardous and the discordant classes; in the aberrant parenting class, depressive symptoms were reduced to similar levels as in the optimal class.

### Pathways from early childhood adversity to adolescent depressive symptoms

**Figure 2.**
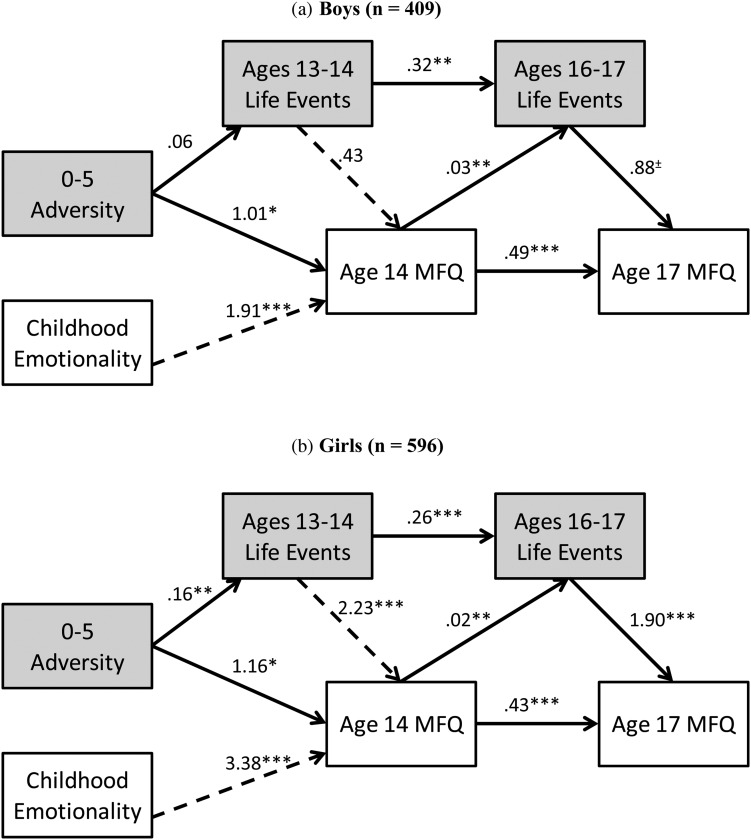
The relationships among early childhood adversity, negative emotionality, age 13 to 14 and 16 to 17 life events, and depressive symptoms at 14 and 17 for (a) boys and (b) girls. Dashed lines indicate a significantly different strength of relationship between the genders. ^±^*p* < .10.

Before conducting path analysis, we characterized the associations between adversity classes, life events, and temperament (see online Supplementary Materials results). In brief, girls reported more life events than boys at 14 and 17 years and parents reported higher levels of negative emotionality in girls. Please see the online Supplementary Materials for details on how the final models were determined. The early childhood adversity models are shown in Figure 2.

#### Boys

For adolescent boys (*n* = 409), as expected from the univariate results, early childhood adversity was significantly related to increased levels of depressive symptoms at age 14 (*p* < .05) but was only indirectly related to age 17 depressive symptoms through age 14 depressive symptoms (β = 0.49, *SE* = 0.22, *p* < .05). There was also a direct path between negative emotional temperament and increases in depressive symptoms at age 14 (*p* < .001) and an indirect path to age 17 depressive symptoms again via age 14 depressive symptoms (β = 0.94, *SE* = 0.28, *p* < .005). However, there was no link between early childhood adversity and life events between ages 13 to 14 (*p* = .40).

Depressive symptoms at 14 years were associated with increased reporting of recent life events at 17 years (*p* < .005), but there were no significant paths from recent life events to depressive symptoms at either 14 or 17 years of age (*p* = .39 and .08, respectively). Negative emotionality was also related to the reporting of life events at 17 years also through age 14 depressive symptoms (β = 0.05, *SE* = 0.02, *p* < .05). Finally, there were no differences in the strength of the relationship between recent life events and depressive symptoms from 14 to 17 years of age (*p* = .24).

#### Girls

For girls (*n* = 596), there was a markedly different and more complex set of associations that involved direct and indirect paths between the psychosocial factors, temperament, and depressive symptoms. Unlike among boys, there was a significant direct link between early childhood adversity and age 13 to 14 life events (*p* < .005), which indicates continuity in adversity from early childhood to early adolescence. In addition, different from boys, both early and late adolescent negative life events significantly predicted subsequent depressive symptoms in adolescent girls (*p*s < .001). There was a significant direct path between early childhood adversity and self-reported depressive symptoms at 14 years (*p* < .05) together with an indirect path via the occurrence of recent life events between 13 and 14 years (β = 0.36, *SE* = 0.14, *p* < .05). Four indirect paths were revealed between early childhood adversity and age 17 depressive symptoms through age 14 depressive symptoms (β = 0.50, *SE* = 0.22, *p* < .05); early adolescent life events and age 14 depressive symptoms (β = 0.16, *SE* = 0.06, *p* < .05); early and late adolescent life events (β = 0.08, *SE* = 0.04, *p* < .05); early adolescent life events, age 14 depressive symptoms, and late adolescent life events (marginal; β = 0.01, *SE* = 0.01, *p* = .05). There was, however, no direct path between early adversity and depressive symptoms at 17 years. Further indirect paths from reported life events between 13 and 14 years of age to depressive symptoms at age 17 were revealed through age 14 depressive symptoms (β = 0.96, *SE* = 0.24, *p* < .001); late adolescent life events (β = 0.49, *SE* = 0.16, *p* < .005); and both age 14 depressive symptoms and late adolescent life events (β = 0.08, *SE* = 0.03, *p* < .05). Early childhood adversity was also related to late adolescent life events through early adolescent life events (β = 0.04, *SE* = 0.02, *p* < .05) and early adolescent life events and age 14 depressive symptoms (β = 0.01, *SE* = 0.003, *p* < .05).

Negative emotionality was directly related to depressive symptoms at 14 years (*p* < .001) and also indirectly related to age 17 depressive symptoms through age 14 depressive symptoms (β = 1.46, *SE* = 0.29, *p* < .001) and age 14 depressive symptoms and late adolescent life events (β = 0.12, *SE* = 0.05, *p* < .05). There was also an indirect path from negative emotionality to life events reported at 17 years through depressive symptoms at 14 years (β = 0.06, *SE* = 0.02 *p* < .01).

The life events and depressive symptoms association did not differ significantly from early to late adolescence (*p* = .61), demonstrating no age-related changes.

#### Gender differences

Two paths leading to depressive symptoms at age 14 were significantly different between the genders (girls > boys): recent life events from 13 to 14 years, χ^2^ (1) = 7.94, *p* < .005, and parent reported emotionality, χ^2^ (1) = 4.61, *p* < .05. However, there was no difference between the genders in the strength of the relationship between late adolescent life events and age 17 depressive symptoms.

#### Interaction effects

We next investigated whether early childhood adversity may increase the depressogenic effect of life events. To test this, we included an interaction term between early childhood adversity and age 13–14 negative life events. For both boys and girls, this interaction was not significant (*p* = .79 and .78), indicating that the effect of life events did not vary depending on early childhood adversity. We further tested whether the effect of negative emotionality and early childhood adversity had interactive effects on predicting depressive symptoms in adolescence. Again, for both genders, this interaction was nonsignificant (*p* = .78 and .48), indicating again the level of negative emotionality and early childhood adversity did not interact to predict a larger depressogenic reaction.

### Pathways from later childhood adversity to adolescent depressive symptoms

**Figure 3.**
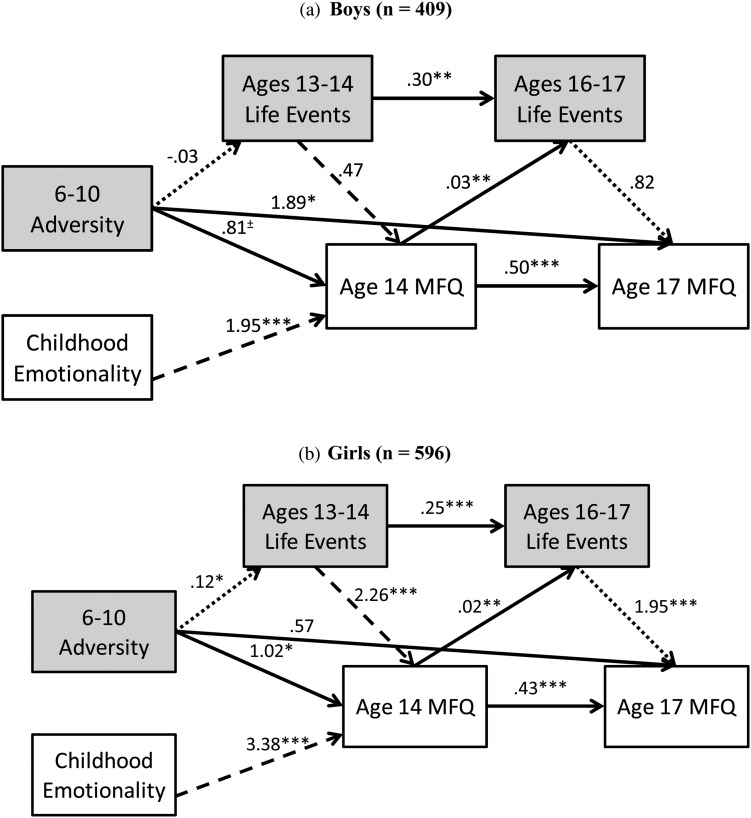
The relationships among later childhood adversity, negative emotionality, ages 13 to 14 and 16 to 17 life events, and depressive symptoms at 14 and 17 for (a) boys and (b) girls. Dashed lines indicate a significantly different strength of relationship between the genders, and dotted lines indicate a marginal difference between the genders. ^±^*p* < .10.

When controlling for early childhood adversities, a similar path analysis emerged, with middle childhood adversity giving similar results for both direct and indirect effects as was found with early childhood adversity (see Figure 3). The main difference with the later childhood path analysis was that there was a direct path between middle childhood adversity and late adolescent depressive symptoms, which was significant only in adolescent boys. As with the early childhood models, interactions terms between later childhood adversity and both age 13 to 14 life events and negative emotionality were not significant in either gender (*p*s > .10).

### Control models excluding recent affective disorders

We next looked at whether our results were being driven by a group of individuals who had experienced a recent episode of an affective disorder, and therefore were likely to have high levels of depressive symptoms. Removing subjects with an episode of an affective disorder between the ages of 13 and 14 or 16 and 17 (*n* = 100) resulted in no substantial differences in the results for the early and later childhood adversity models, with the exception of the significant link between later childhood adversity and early adolescent depressive symptoms, which became nonsignificant in adolescent girls (*p* = .13; models available from the first author).

### Childhood adversity class specificity

Relative contributions of each class were examined (see online Supplementary Materials) to determine if the overall model effects were driven by distinct subtypes. For early adversities, discordant and hazardous subtypes were strongest in driving the models for boys and girls, respectively. The aberrant parenting subtype did not appear to contribute to depressive symptom reporting for either gender. This model was broadly retained for both genders when considering later childhood adversity subtypes, except for girls where, the aberrant class was contributing to depressive symptom reporting at 14 years.

## Discussion

The first objective in this paper was to test the likelihood of direct (enduring type) versus indirect (revisionist type) models linking childhood adversities to adolescent depressive symptoms. Overall, there was evidence across adolescent girls and boys supporting both models dependent on age. The findings demonstrated the value of categorizing timing of exposure to adversities as well as class membership. This was apparent in the finding that early and later childhood exposures had a differential effect by class on the likelihood of depressive symptoms at 14 and 17 years. We also found that childhood adversities maintain an environment in which proximal negative events are more likely to be reported, but only in girls. There were also gender differences with regard to the impact of proximal life events. In girls but not boys, proximal life events were found to at least partially moderate the direct effect of childhood adversity on late adolescent depressive symptoms. These findings provide some novel insights into gender-differentiated sensitivity to the environment, the role of timing and maturation on associations with adversities, the impact of emotionality on depressive symptoms over time, and the potential bidirectional effects between social and cognitive systems in the adolescent years.

### Latent classes reveal how childhood adversities may exert their effects

The depressive symptom reporting at 14 years of age in the 47% of the cohort exposed to some type of childhood adversity did not follow a simple relationship but did show clear-cut gender differences. For both boy and girl adolescents, only those in the most severe hazardous adversity subtype (consisting of 6% of the sample) in the preschool years had any marked degree of influence on the level of depressive symptoms at the age of 14. Only for girls in this hazardous class, however, was there an enduring path with an effect on depressive symptoms at 17 as well as at 14 years of age. For boys, even the impact of an exceptionally hazardous early childhood environment dissipates across adolescence, with the increased levels of depressive symptoms found at age 14 reducing by age 17, suggesting effects if present may operate via a social revisionist type model or reduce through maturation effects on the initial negative cognitions (Fraley et al., 2013; Roisman et al., 2009).

These findings support the first hypothesis, that LCA approaches add value by identifying at the population level an empirical grouping of 6% of the participants at most risk for elevated depressive symptoms by 14 years of age following exposure to hazardous adversities in the preschool years. The findings are also consistent with the long-standing evidence that both severe marital discord and direct maltreatment to the preschool child carries long-term depressogenic effects throughout adolescence. However, in this study this appears to be uniquely for girls. What occurs in the environment or within boys to reduce such an effect deserves further investigation.

In contrast, membership of aberrant parenting and discordant adversity subtypes in the preschool years appear to be less depressogenic overall for both genders than previously considered from cross-sectional family studies. The findings do suggest that gender-differentiated latent mechanisms may be operating following exposure to distinctive qualities of suboptimal family environments in the childhood years. It is interesting that, taking the results as a whole, there is no support for a simple linear severity or dose–response model of the impact of childhood adversities on depressive symptom reporting at 14 and 17 years.

### Maturational influences on the impact of the environment and depressive symptoms

Within these findings, there is some evidence to support the second hypothesis of a maturational effect lowering the impact of early childhood adversities on depressive symptoms by 17 years but in boys only. The causes of such developmental effects require further investigation. For girls, however, there was no support for a latent maturational effect on the impact of early childhood adversity on early or later adolescent depressive symptoms. One note of caution is that this gender difference may be explained by a heterotypic outcome by 17 years. Thus, the enduring effects of hazardous preschool experiences may increase higher levels of nondepressive behavioral symptoms in later adolescence in boys compared to girls (Moffitt & Caspi, 2001). This might indicate a transdiagnostic mechanism arising early in development as a consequence of exposure to childhood adversities, which is apparent in this cohort (Dunn et al., 2011).

There are also putative effects dependent on the timing of adversity, in support of the second hypothesis. Specifically, there are rather different findings for symptom reporting to later childhood adversities at 14 years. Here a greater number of adversity subtypes were associated with increased depressive symptoms, suggesting that the more proximal experiences in the primary school years result in more depressogenic responses among the subtypes in adolescence, consistent with previous observations (Shanahan et al., 2011).

The response in early adolescence by both genders to adversities in the primary school years may reflect a growing understanding of the personal implications of threats to family well-being. There are again, however, observable gender differences. For boys, both the hazardous and the discordant classes were associated with elevated symptoms at 14 years. However, for girls there were increased depressive symptoms at age 14 for all three adversity subtypes. It may be that girls have a lower threshold of sensitivity or boys are somewhat more resilient to milder aberrant parenting adversities from both parents. By 17 years, for both genders, the putative effects of exposure to adversities in the primary school years remained for the hazardous class only, supporting a persistent enduring-type effect model in both genders in this class only. This suggests that maturation and/or timing effects are insufficient to reduce negative consequences for boys and girls who experienced hazardous adversity in the primary school years. There is some evidence in support of an indirect revisionist-type and/or maturation effects model to milder aberrant parenting in adolescent girls, with the effect of this class only shown at age 14 not at age 17. Further research could illuminate other factors that may account for this reduction in adolescent girls.

### Pathways to depressive symptom formation from childhood through to adolescence

Using the path analytic techniques further emphasizes gender differences in the longitudinal associations existing from childhood adversities to emerging depressive symptoms by 17 years of age. For boys, there were significant but indirect paths from both early childhood adversities and negative emotional temperament to depressive symptoms at 17 years. This pattern was also found in adolescent girls although with additional direct and indirect paths through proximal negative life events. In contrast, later childhood adversities showed a main and more enduring-type effect on depressive symptoms at age 17 for adolescent boys, consistent perhaps with the impact of maturation on cognition as suggested above. This is especially striking when contrasted with the pattern for adolescent girls, where all paths for adolescents from both early and later childhood adversities show indirect paths to emerging depressive symptoms by 17 years. Overall, the findings support the fourth hypothesis, that there are gender-differentiated pathways accounting for the effects of childhood adversities on adolescent depressive symptom formation.

### Gender differences in the role of proximal life events during adolescence

While it is well recognized that girls report more recent negative life events, the current findings go further in elucidating the putative gender-differentiated role of such events in the emergence of depressive symptoms by later adolescence. For adolescent boys, there is a clear noncausal effect of proximal negative events on depressive symptoms, which is consistent with previous work (Rudolph & Flynn, 2007), although depressive symptoms in early adolescent boys do relate to elevated negative events in late adolescence. These patterns indicate that for 14-year-old boys, early childhood adversity is associated with depressive symptoms by early adolescence and that retrospective life event reporting in late adolescence is at least partly depression symptom dependent. In contrast, for girls there were multiple paths to elevated depressive symptoms over adolescence involving the bidirectional effects between life event and depressive symptom reporting at 14 and 17 years. It seems that there is a much more permissive set of paths to depressive symptoms by the end of adolescence for girls compared to boys. In addition, it is clear that childhood adversity increases the likelihood of life events, which in turn lead to increases in depressive symptom in girls only. Thus, early adversity appears to set in action a chaining of effects that increase the likelihood of more proximal risk factors in adolescent girls.

### Emotionality and depressive symptoms

For both genders, the childhood disposition of higher emotionality is associated directly with depressive symptoms at 14 years and indirectly (through symptoms) to depressive symptoms at 17 years. In girls only, however, there were two further components: first, the relationship between negative emotional temperament and age 14 depressive symptoms was significantly stronger; and second; there are indirect paths between this emotion disposition and proximal life event reporting at 17 years. Similar to early adversity, we find that the distal risk factor of negative emotionality is contributing to maintenance of more proximal negative life events leading to increased depressive symptoms in adolescent girls, namely, proximal life events. Thus, the increased influence of negative emotional temperament likely contributes to the well-established finding of higher depressive symptoms in adolescent girls (Euling et al., 2008; St Clair et al., 2012) as well as potentially contributes to the higher rate of negative life events in girls as well, which then also increases depressive symptoms.

For both genders, however, the findings emphasize that care must be taken when attributing a direct causal role of child maltreatment to mental illnesses that emerge in adult life because the intervening years may be the source of critical mediating processes for later psychopathology and the direction of effects between life event and symptom reporting may be different between the genders.

### Maturing cognition during adolescence and emerging depressive symptoms

The aforementioned findings also suggest that neurodevelopmental maturing cognitive systems are likely to be important substrates for differential sensitivities to the environment. Furthermore, depressive symptoms themselves appear to be potentially influential in shaping these systems because in both genders there are consistent paths to depressive symptoms by 17 and positive life event-depression symptom reporting associations for both genders. Of importance, the current findings do not support the notion of a narrow sensitive period where those exposed to a suboptimal family adversity develop a fixed and vulnerable cognition that is enduring and persistent. This resonates with Jaffee and Maikovich-Fong (2011), who reported elevated emotional and behavioral difficulties in children exposed to chronic and severe maltreatment over the first 10 years of life with little specificity in the timing of negative experiences. The current findings provide a novel observation that suggests it is the school-age years, when contrasted with the preschool years, which appear to be a more sensitive time for exposure to milder forms of adversity, or suboptimal family environments. This implies an important policy strategy of improving the connections between family and primary school environments as being a key mediator in the development and maintenance of subsequent good adolescent mental health for both genders.

### Limitations

As well as depressive symptoms, exposure to childhood family adversities exerts nonspecific risks for a range of psychopathologies throughout adolescence into young adult life (Green et al., 2010; Shanahan et al., 2011). The extent, however, to which the psychosocial pathways described here also relate to other symptom profiles (e.g., anxiety, antisocial behavior, obsessive–compulsive, and psychotic-like symptoms) remains to be explored.

Life events were self-reported, allowing for the possibility that adolescent girls may be more willing to report negative life events than are boys. In addition, depressed adolescents may perceive events in a more negative manner, which may also increase the gender difference. However, gender differences in the occurrence of life events have also been demonstrated with interview methods using independent, external scoring (Leadbeater, Kuperminc, Hertzog, & Blatt, 1999; Shih, Eberhart, Hammen, & Brennan, 2006). We consider reporting bias possible, but unlikely. Parents may also have underreported overt maltreatment or abusive experiences. The use of the latent classes, which utilized a contingent of multiple correlated adversities in determining the adversity classes, is likely to have compensated in part for the lack of reporting severe abuse by the reporting of less severe but correlated adversities. However, more accurate reporting would likely only have strengthened the pathways outlined in this paper. It may also be that reporting adversity in childhood may influence the parents' perceptions of the child's current functioning and influence the reporting of negative emotionality. This seems to be unlikely, given that there was no consistent relationship between negative emotionality and the suboptimal adversity classes (see online Supplementary Materials).

The measure of negative emotionality may also have been biased by more current behavior of the adolescents. Negative emotionality, like all temperament traits, has been found to be remarkably stable developmentally, and therefore we view our measurement as a good indication of childhood temperament. Within this sample, we have found this measure to be remarkably stable across adolescence (Spence, Owens, & Goodyer, 2013). Nevertheless, we cannot discount that recent behavior or emotional problems may have influenced the parental rating of how their child generally behaves. We did evaluate how this measure was related to current mood at age 14. Depressive symptoms accounted for only 4% in boys and 9% in girls of the variation in negative emotionality, which indicates a significant but weak relationship between these two factors unlikely to undermine the current results (analysis available from first author).

## Conclusions

This study provides prognostic validity for a latent class approach to define empirically determined groups of adolescents exposed to correlated family adversities in two periods in childhood, 0–5 and 6–11 years, respectively. Whether there is a direct enduring-type or an indirect revisionist-type set of effects of childhood adversities is determined by adversity class membership, exposure in preschool or school-age years, and gender. Population stratification of adolescents by childhood adversity classes would increase the opportunity for delineating distinctive mechanism arising from exposure to differing forms of childhood adversity in the preschool and/or primary school years. There are also gender-differentiated mechanisms by which intervening factors (negative life events in adolescents) in part modulate the direct effect of childhood adversity to later outcomes. Thus, following exposure to mild to moderate childhood adversities, there may be key gender-differentiated latent neurodevelopmental factors that emerge in adolescence that reduce depressogenic symptoms in boys but increase such formation in girls. Greater attention to family environments in the primary school years for both girls and boys could, however, potentially reduce the emergence of depressive symptoms in adolescents and secondary schools.

## Supplementary Materials

The online only Supplementary Materials can be found online at http://journals.cambridge.org/dpp
